# Progression of Spinal Cord Disease in Adult Men With Adrenoleukodystrophy

**DOI:** 10.1002/jimd.12845

**Published:** 2025-01-07

**Authors:** Hemmo A. F. Yska, Marije Voermans, Eda Kabak, Marc Engelen

**Affiliations:** ^1^ Department of Neurology and Pediatric Neurology, Emma Children's Hospital, Amsterdam Leukodystrophy Center Amsterdam University Medical Center Amsterdam The Netherlands

**Keywords:** Dutch ALD cohort, leukodystrophy, natural history, trial

## Abstract

This study presents the longest systematic prospective follow‐up of spinal cord disease in adult male ALD patients to date. Standardized yearly quantitative data collection included scoring of the EDSS, SSPROM, 6‐min walking test (6MWT), urological and quality of life questionnaires and vibration sense of the hallux. Progression rates were compared between patients with mild (EDSS ≤ 2.5) and moderate to severe (EDSS > 2.5) disability over a period of 7 years. Data from 79 patients was included. EDSS, SSPROM and 6MWT showed significant disease progression over time. The general progression pattern was linear. Stratified by disease severity, the increase in EDSS was 0.1 points per year in the low EDSS group and 0.2 points per year in the higher EDSS group. SSPROM decreased by −0.7 points per year in the low EDSS group and by −1.9 points per year in the higher EDSS group. 6MWT decreased by −9.3 m/year in the low EDSS group and by −18.2 m/year in the higher EDSS group. The rate of progression in patients with relatively severe disability was higher than in patients with mild disability. Clinical trials will therefore observe effects more rapidly in patients with more advanced disease.

## 1 Introduction

Adrenoleukodystrophy (ALD) is a neurometabolic disease caused by pathogenic variants in the *ABCD1* gene [1]. Two main manifestations are recognized in the central nervous system: a rapidly progressive leukodystrophy often referred to as cerebral ALD (cALD) and a slowly progressive spinal cord disease called adrenomyeloneuropathy (AMN). All adult males and most adult females will develop spinal cord disease. About 60% of male patients develop leukodystrophy [2]. The spinal cord disease of ALD is characterized by progressive axonal loss in predominantly the corticospinal tracts and dorsal columns and the peripheral nerves [3, 4, 5, 6]. Core symptoms are a gait disorder, sensory symptoms and bladder‐ and bowel dysfunction [6, 7, 8]. Age of onset and rate of progression are highly variable. Disease severity in an individual patient can currently not be predicted (for instance, genotype–phenotype correlation could not be established) [9]. However, it was shown recently that the level of plasma C26:0‐lysophosphatidyl choline (C26:0‐LPC) correlates with disease severity and explains part of the variability at group level [10].

Quantitative data on long‐term disease progression is essential for the design of clinical trials, specifically for outcome measure selection and duration of the trial. Monitoring the progression of the spinal cord disease is complicated by the slow progression, making it difficult to detect change on clinical outcome measures as well as surrogate outcome measures [11]. For instance, spinal cord atrophy is correlated to disease severity but becomes apparent only in advanced disease [12]. Clinical outcome measures such as the 6‐min walking test (6MWT) and the Expanded Disability Scoring Scale (EDSS) remain commonly used but are not very sensitive or specific for changes over time [11]. The “Dutch ALD cohort” is a prospective natural history study that was initiated in 2015 and is still ongoing. Its main goal is to collect quantitative data on the progression of spinal cord disease. Previous papers have reported on progression of spinal cord disease over 2 years, incidence of adrenal failure, and potential novel outcome measures [12, 13, 14, 15, 16, 17]. This study reports progression of the spinal cord disease over a period of 8 years for clinical parameters. The goals of this study are to (1) report the prevalence of signs and symptom of spinal cord disease in adult men with ALD as related to age, (2) describe the rate and pattern of progression in adult men with ALD. This data is important for counseling patients and will help to design future therapeutic trials.

## 2 Materials and Methods

### 2.1 Participants

This study was approved by the local Institutional review board (#2018_310). The Amsterdam University Medical Center is the main referral center for ALD in the Netherlands and provides care for virtually all ALD patients in the Netherlands. Male patients with ALD (confirmed by genetic and biochemical testing) that provide written informed consent are included in the Dutch ALD cohort and visits are planned once a year. Currently, more than 100 male patients have been included. For this study, we analyzed data collected between 2015 and 2023 (maximum of 8 time points). For the cross‐sectional analyses, participants were stratified by age: < 30 years, 30–50 years and > 50 years. These categories were chosen based on previous publications [11].

## 3 Outcomes

### 3.1 Neurological Examination

A standardized neurological examination was performed by a trained physician as described previously [11]. Vibration sense was quantified with a Rydel–Seiffer tuning fork (0 (absent)–8 (normal)). A score of 6 or lower was considered as diminished vibration sense [18]. The presence of a gait disorder was qualitatively assessed by clinical observation (normal or abnormal). A full neurological examination was performed, but the current work only describes results of the lower extremities as results for the upper extremities were generally normal. Patients were categorized as having a myelopathy if they had both signs and symptoms of spinal cord disease [11, 19].

### 3.2 Adrenal Insufficiency

Adrenal insufficiency was considered present if patients used glucocorticoid or mineralocorticoid suppletion therapy.

### 3.3 Bladder and Bowel Dysfunction

Patients were asked if they were incontinent for urine or feces. Patients were also queried on libido and erectile dysfunction. Symptoms were quantified using the International Consultation on Incontinence Questionnaire Male Lower Urinary Tract Symptoms Module (ICIQ‐MLUTS) [20]. Separate scores were calculated for voiding and incontinence, where higher scores reflect more severe complaints.

### 3.4 Clinical Scores

Disease severity was quantitatively assessed by four functional outcomes: 6MWT [21], EDSS [22], Severity Score System for Progressive Myelopathy (SSPROM) [23], and timed‐up‐and‐go (TUG) [24]. Data collection was performed as previously described [11]. In accordance with definitions, patients with an EDSS ≤ 2.5 were considered to have minimal disability and patients with an EDSS > 2.5 were considered to have moderate to severe disability [25].

### 3.5 Quality of Life

Patients were asked whether they had visited a psychologist or psychiatrist in the past year. General quality of life was quantified by the short‐form health (SF‐36) questionnaire, which consists of eight domains [26]. In the analyses, emphasis was placed on the physical functioning and physical health domains of the questionnaire as they were previously found to be most affected in ALD [19]. Participants were also asked whether they lived independently or required help.

### 3.6 Statistical Analysis

Means and standard deviations were used to describe the baseline data. A Kaplan–Meier curve was created to visualize the proportion of patients without a myelopathy in relation to age. Disease progression was assessed by means of (1) EDSS, (2) SSPROM, (3) 6MWT, (4) ICIQ‐MLUTS voiding and incontinence scores, (5) SF36 physical and physical health subscores and (6) vibration sense on the mean of both halluces measured with a Rydell–Seiffer tuning fork. Mixed linear models were used to assess progression over time. Longitudinal group estimated means were visualized and compared between patients with minimal disability (EDSS ≤ 2.5) and patients with moderate to severe disability (EDSS > 2.5) at baseline. The minimal disability group also includes asymptomatic patients at baseline (EDSS = 0). As an exploratory analysis, analyses were repeated without these individuals. Time, EDSS category (minimal vs. moderate to severe disability), age at first visit and an interaction term (time*EDSS category) were included as fixed factors in the models with age at baseline as a covariate. Patient ID was included as a random effect. An alpha level of 0.05 was considered statistically significant.

## 4 Results

A total of 79 adult males were included in the analysis of this ongoing natural history study with follow‐up of up to 7 years (Table 1). Fifteen patients (19%) withdrew from this study for various reasons. Two patients developed a leukodystrophy and treatment was transferred to a different hospital. Five patients dropped out for medical reasons not related to ALD. One patient indicated that our follow‐up protocol resulted in too much anticipatory stress. Invalidating pain related to ALD prohibited one patients from physically coming to the hospital. Six individuals were lost to follow‐up. The mean time between visits was 13 months.

**TABLE 1:** Number of participants per visit.

Visit no	Participants (*n*)
1 (baseline)	79
2	65
3	50
4	44
5	37
6	34
7	27
8	17

### 4.1 Cross‐Sectional Data

**FIGURE 1:**
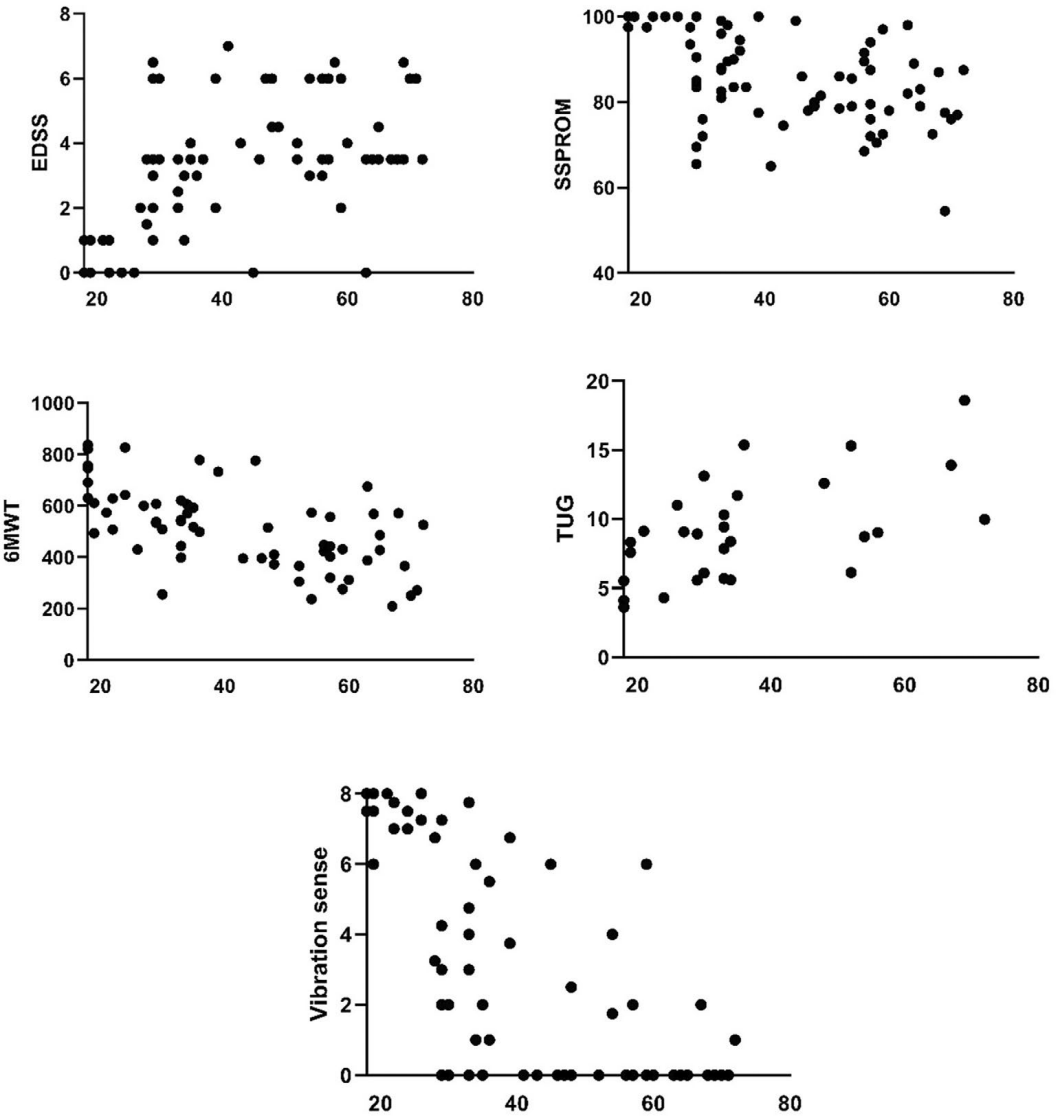
Quantitative outcomes at baseline. Age in years at baseline is presented on the *X*‐axis. Vibration sense hallux represents the mean of both halluces. Worse disease severity is suggested by higher values for EDSS, TUG and ICIQ‐MLUTS subscores and lower values for SSPROM, 6MWT, vibration sense and SF‐36 subscores. 6MWT: 6‐min walking test; EDSS: Expanded Disability Scoring Scale; ICIQ‐MLUTS: International Consultation on Incontinence Questionnaire Male Lower Urinary Tract Symptoms Module; SF‐36: Short‐Form health survey; SSPROM: Severity Score System for Progressive Myelopathy.

At their inclusion visit, the majority of patients (*n* = 57, 72%) presented with a myelopathy. Table 2 describes baseline data for all participants stratified by age at inclusion. Table S1 shows the same information stratified by EDSS at inclusion. Figure 1 shows quantitative metrics as a function of age at baseline. The higher age categories had a higher prevalence of myelopathy. None of the older participants in this cohort were wheelchair dependent and only one patient relied on help of others in daily activities. Incontinence for feces was present at about the same level for all age groups. The EDSS and TUG were higher whereas SSPROM and 6MWT were lower in older than in younger patients, reflecting more severe disability. All SF‐36 subscores in the oldest age group were lower than in the Dutch reference population, reflecting a higher perceived disease burden [26]. In contrast, at their baseline visit, younger patients scored above average on the physical health, physical function, emotional well‐being and pain subscores. The largest proportion of patients who indicated they visited a psychologist or psychiatrist was in the middle age category. Use of adrenal suppletion therapy was lower in older than in younger individuals.

**TABLE 2:** Background of participants at baseline.

	< 30 years (*n* = 28)		30–50 years (*n* = 23)		> 50 years (*n* = 28)	
General	*n*		*n*		*n*	
Age (years)	28	24.1 (4.7)	23	39.0 (5.8)	28	61.0 (6.1)
Male balding	27	13 (48%)	23	21 (91%)	28	24 (86%)
Glucocorticoid insufficiency	28	23 (82%)	23	10 (44%)	28	7 (25%)
Mineralocorticoid insufficiency	28	11 (39%)	23	5 (22%)	28	3 (11%)
Myelopathy	28	10 (36%)	23	19 (83%)	28	28 (100%)
Visited psychologist in past year	27	4 (15%)	21	5 (24%)	28	2 (7%)
Neurological examination						
Pathological reflexes	27	12 (43%)	23	18 (78%)	28	26 (93%)
Decreased strength (MRC < 5)	28	4 (14%)	23	6 (26%)	28	11 (39%)
Leg spasticity	28	7 (25%)	23	10 (44%)	28	14 (50%)
Decreased propriocepsis	26	5 (19%)	19	11 (58%)	27	16 (59%)
Decreased warm‐cold discrimination sense	24	4 (17%)	17	6 (35%)	22	10 (46%)
Postural instability	27	7 (26%)	23	16 (70%)	27	23 (85%)
Degree of disability						
Gait disorder	28	8 (29%)	23	16 (70%)	28	23 (82%)
Falling/tripping	28	8 (29%)	23	12 (52%)	28	24 (86%)
Walking with aid	28	3 (11%)	23	4 (17%)	28	10 (36%)
Use of wheelchair	28	0 (0%)	23	1 (4%)	28	0 (0%)
Independence in daily activities	28	1 (4%)	23	0 (0%)	28	1 (4%)
Urogenital issues						
Incontinence urine	27	6 (22%)	23	8 (35%)	28	12 (43%)
Libido problems	17	3 (18%)	17	5 (29%)	25	14 (56%)
Erectile dysfunction	18	5 (28%)	18	8 (44%)	26	15 (58%)
Incontinence feces	28	6 (21%)	23	5 (22%)	28	5 (18%)
Quantitative measures						
EDSS	28	1.7 (2.0)	23	3.5 (1.7)	28	4.3 (1.6)
SSPROM	28	93.6 (11.0)	23	86.3 (9.0)	28	81.0 (9.5)
6MWT (meters)	20	612.2 (144.4)	18	539.5 (129.1)	24	409.9 (124.7)
TUG (seconds)	13	7.4 (2.9)	9	9.7 (3.2)	7	11.7 (4.4)
SF36 physical functioning	20	85.0 (26.5)	22	67.3 (26.5)	25	50.2 (25.8)
SF36 physical health	23	80.4 (34.5)	23	79.3 (36.7)	26	54.8 (45.8)
SF36 emotional problems	24	86.1 (32.5)	23	84.1 (29.9)	26	76.9 (40.9)
SF36 energy/fatigue	24	63.3 (16.7)	23	51.7 (18.1)	26	55.4 (23.7)
SF36 well‐being	24	68.8 (20.7)	23	62.1 (23.9)	26	70.3 (24.3)
SF36 social functioning	24	78.4 (21.4)	23	67.6 (24.2)	26	62.6 (23.6)
SF36 pain	24	88.3 (15.7)	23	79.0 (23.0)	26	68.0 (25.6)
SF36 average health	24	61.5 (20.2)	23	50.0 (18.6)	26	55.6 (21.5)
ICIQ‐MLUTS‐ voiding	19	5.2 (5.0)	22	5.8 (5.2)	26	8.5 (5.2)
ICIQ‐MLUTS‐incontinence	19	3.2 (3.4)	22	3.1 (2.9)	26	5.6 (5.1)
Vibration sense hallux	26	5.8 (2.8)	21	2.9 (2.8)	23	0.6 (1.4)

*Note:* Data is presented in three age categories as mean and (standard deviation) or as number of patients and (percentage).

Abbreviations: 6MWT: 6‐min walking test; EDSS: Expanded Disability Scoring Scale; ICIQ‐MLUTS: International Consultation on Incontinence Questionnaire Male Lower Urinary Tract Symptoms Module; SF‐36: Short‐Form health survey; SSPROM: Severity Score System for Progressive Myelopathy; TUG: timed‐up‐and‐go.

In most patients with a myelopathy (*n* = 49, 86%), a combination of motor and sensory signs and symptoms was observed as the presenting feature. Two patients (4%) presented with only motor fatigability, five patients (7%) presented with decreased position sense or vibration sense and one patient (2%) presented with decreased discrimination sense. Two additional patients in the asymptomatic group developed a myelopathy during follow‐up. Decreased position sense and vibration sense were the presenting signs in both.

### 4.2 Longitudinal Data

**FIGURE 2:**
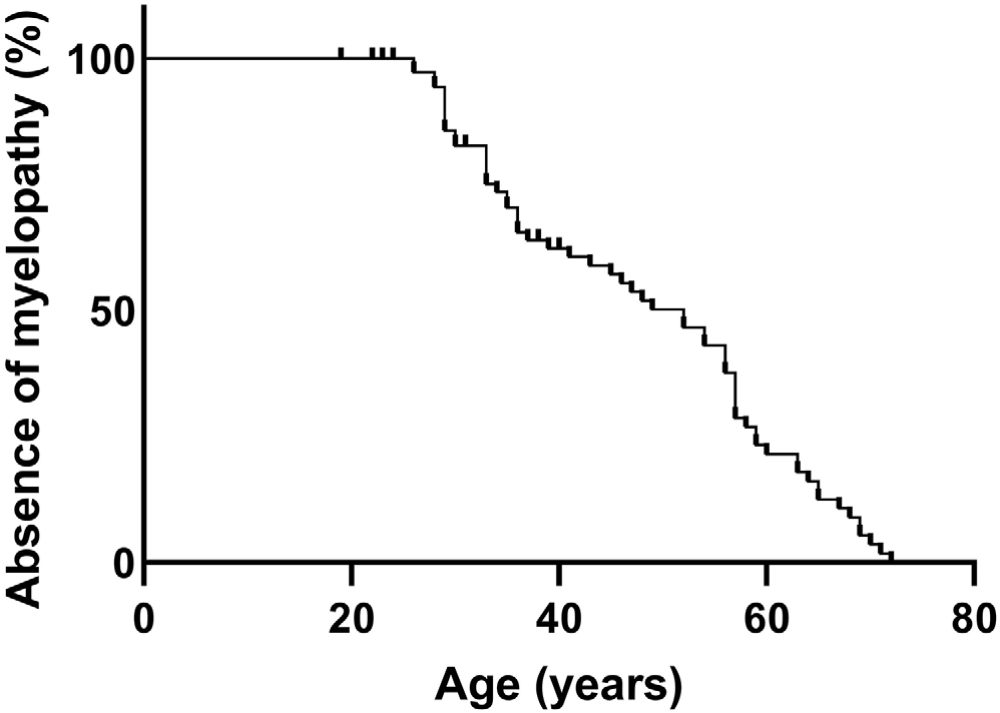
Development of myelopathy. The absence of myelopathy is presented as a function of age. Nineteen patients who had not yet developed a myelopathy were censored at their last visit.

Figure 2 shows the presence of myelopathy as a function of age. A total of 19 patients (24%) had not (yet) developed a myelopathy during follow‐up and were therefore censored at the age of their last visit. At 50 years of age, approximately 50% of patients had signs and symptoms consistent with a myelopathy.

**FIGURE 3:**
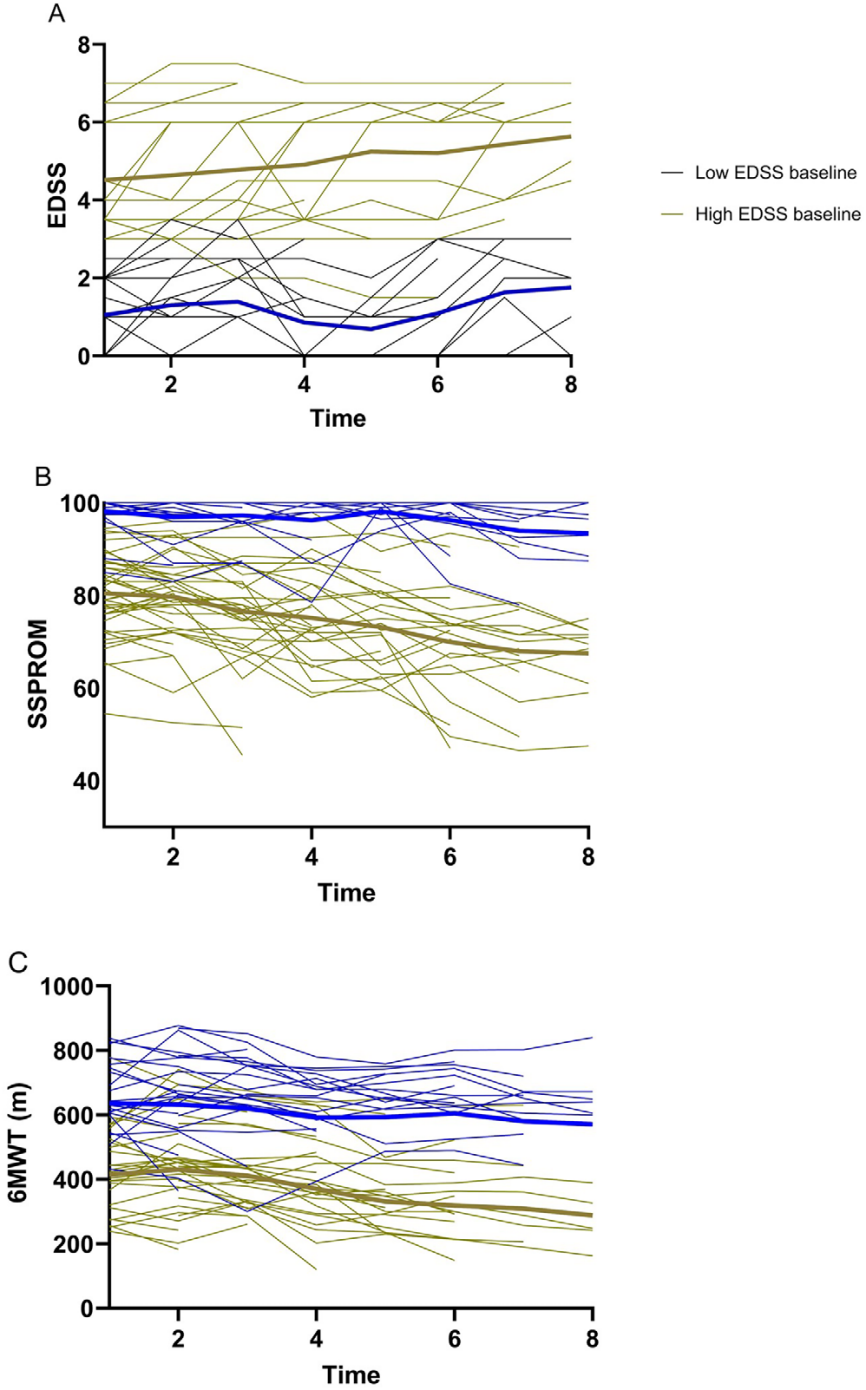
Progression of EDSS, SSPROM and 6MWT over time. *X*‐axis: Visit 1 represents baseline visit. Visits were separated by a mean interval of 13 months. Low EDSS: EDSS ≤ 2.5; high EDSS: EDSS > 2.5. Bold black lines represent estimated means. 6MWT: 6‐min walking test; EDSS: Expanded Disability Scoring Scale; SSPROM: Severity Score System for Progressive Myelopathy.

Estimated means generated by mixed linear models for all variables are presented in Table 3. EDSS increased and SSPROM and 6MWT decreased significantly (*p* < 0.01) over time (Figure 3). All three parameters generally showed mean linear progression. For the 6MWT, 13 patients did not show worsening of walking distance during follow‐up. A significant effect was also found for vibration sense on the hallux (*p* = 0.035) but estimated means increased as opposed to the expected decrease (from baseline to *t* = 7: 3.3 to 3.5). No significant effects were observed for the ICIQ‐MLUTS or for the subscales of the SF‐36. Based on the estimated means, the mean increase in EDSS between inclusion and *t* = 7 was 0.9 points (0.1/year), the mean decrease in SSPROM was −9.0 points (1.3/year) and the mean decrease in 6MWT was −97.3 m (13.9/year). The slope for the change of the estimated means for EDSS, SSPROM and 6MWT of patients with the lower EDSS scores at baseline was less steep than for patients with higher EDSS scores. When split for groups, changes for EDSS were 0.7 (0.1/year) points in the low EDSS group and 1.1 (0.2/year) points in the high EDSS group. SSPROM decreased by −4.8 (−0.7/year) points in the low EDSS group and by −13.0 (−1.9/year) points in the high EDSS group. 6MWT decreased by −65.2 (−9.3/year) meters in the low EDSS group and by −127.1 (−18.2/year) meters in the high EDSS group. Exclusion of asymptomatic individuals in the low EDSS group still resulted in slower progression compared to the high EDSS group.

**TABLE 3:** Mean progression of quantitative outcome measures.

Time	1	2	3	4	5	6	7	8
EDSS	2.9 (0.2)	3.1 (0.2)	3.3 (0.2)	3.1 (0.2)	3.2 (0.2)	3.4 (0.2)	3.7 (0.2)	3.9 (0.2)
SSPROM	88.6 (1.1)	87.5 (1.1)	86.1 (1.2)	84.9 (1.2)	84.8 (1.2)	82.3 (1.3)	80.1 (1.3)	79.6 (1.5)
6MWT (meters)	518 (20)	523 (20)	507 (20)	473 (21)	454 (21)	454 (21)	437 (22)	421 (24)
Vibration sense hallux	3.3 (0.3)	3.3 (0.3)	3.0 (0.3)	3.3 (0.3)	3.7 (0.4)	4.0 (0.4)	3.8 (0.4)	3.4 (0.4)
SF‐36 (physical health)	75.6 (5.9)	70.8 (6.1)	69.8 (6.3)	72.5 (6.5)	68.5 (7.0)	74.0 (7.3)	67.8 (8.1)	76.3 (10.3)
SF‐36 (physical functioning)	68.0 (3.9)	68.2 (3.9)	65.8 (4.0)	65.8 (4.1)	66.9 (4.3)	69.8 (5.3)	65.2 (4.8)	57.2 (5.3)
ICIQ‐MLUTS (incontinence)	3.2 (0.7)	3.3 (0.7)	3.5 (0.7)	3.5 (0.7)	3.3 (0.7)	3.3 (0.7)	3.8 (0.8)	2.7 (0.8)
ICIQ‐MLUTS (voiding)	6.3 (0.7)	5.7 (0.7)	6.2 (0.7)	6.3 (0.7)	6.7 (0.7)	6.1 (0.7)	6.5 (0.8)	5.5 (0.9)

*Note:* Data is presented as estimated mean and (standard error). Vibration sense hallux represents the mean of both halluces.

Abbreviations: 6MWT: 6‐min walking test; EDSS: Expanded Disability Scoring Scale; ICIQ‐MLUTS: International Consultation on Incontinence Questionnaire Male Lower Urinary Tract Symptoms Module; SF‐36: Short‐Form health survey; SSPROM: Severity Score System for Progressive Myelopathy.

## 5 Discussion

Quantitative data on the symptoms and progression of ALD spinal cord disease is of great importance for counseling patients and for design of clinical trials. This study presents the longest systematic prospective follow‐up of male ALD patients to date and provides novel data and insights. Linear mixed models showed that the mean EDSS, SSPROM and 6MWT changed significantly over time in an approximately linear fashion. Some patients had fluctuating scores over time, which reflects limitations and inaccuracies of these specific outcomes. The 6MWT, for example, is known to be influenced by motivation and use of a walking aid [27, 28]. Huffnagel et al. identified a mean change of 0.34 points per year for the EDSS, a change of −2.78 points per year for the SSPROM and a change of −19.67 m/year for the 6MWT in symptomatic males with ALD spinal cord disease [11]. The observed changes in the current study are lower (EDSS: 0.1/year, SSPROM: −1.3/year, 6MWT: −13.9/year). An explanation for this discrepancy may be that more relatively healthy individuals participated in this study, as illustrated by the fact that only one individual used a wheelchair at baseline. Presymptomatic or mildly affected individuals were included in the cohort after extended family screening and this larger cohort likely better reflects the ALD population as a whole. Strikingly, the progression of the EDSS, SSPROM and 6MWT differed clearly between patients with low and high EDSS scores at baseline. This is most likely because individuals with early‐stage disease have more compensatory reserve and is likely not related to a difference in the rate of axonal degeneration. These findings are useful for the design of clinical trials. The data shows that clinical trials using the outcomes described in this paper that include asymptomatic or early disease stage patients will likely not detect change over several years. In clinical practice, patients with EDSS scores > 2.5 can be counseled that their disease burden will increase more rapidly than in earlier stages of disease. This may, for example, lead to preventive in‐house accessibility adjustments or more frequent hospital visits. Conversely, patients with EDSS scores ≤ 2.5 may benefit from less frequent hospital visits as it is unlikely that symptom severity will rapidly increase.

In contrast to other outcomes, SF‐36 physical and physical health scores and ICIQ‐MLUTS scores did not change significantly over time. Patient‐reported outcomes are increasingly popular in medical research and provide a more central role for patients [29]. Little research has been performed on the effect of ALD spinal cord disease on mental health [30]. The results presented here suggest that, although SF‐36 scores worsen over time and are lower than Dutch reference populations, change is non‐significant over a period of about 7 years. This indicates that the SF‐36 is not a sensitive outcome measure to evaluate the effects of an intervention on spinal cord disease progression. However, for trials with interventions aimed at increasing general well‐being the SF‐36 could be an appropriate endpoint. The significant but generally stable results for vibration sense on the hallux contrast to previous studies [8, 11]. In this study, vibration sense was measured with a Rydell‐Seiffel tuning fork, which is semi‐quantitative and somewhat subjective. Some patients demonstrated inconsistent results between years, which illustrates that intra‐patient variability is high. Keller et al. made use of a “Vibraton”, which measures vibration sense on a more continuous scale and could therefore be more reliable. Alternatively, severely affected patients may have reached the lowest possible value of “0” thus preventing further progression in this long‐term study.

The age that 50% of patients had developed a myelopathy was higher (around 50 years) than previously reported by Huffnagel et al. (around 40 years) [11]. This may be related to the larger population studied that now includes more early‐stage patients, due to increased diagnosis rate compared to the past because of the introduction of Whole Exome Sequencing and newborn screening. Abnormal vibration and position sense were common presenting signs of myelopathy and were observed in 11%. Two additional patients converted to a myelopathy during follow‐up and also presented with these abnormalities. In line with pathological studies, this suggests the early involvement of the dorsal columns in ALD spinal cord disease [31, 32].

A number of cross‐sectional findings requires attention. At their inclusion visit, glucocorticoid and mineralocorticoid suppletion therapy were less common in older than in younger patients. This may be because patients who were included in this study at an older age were more likely to have been diagnosed after experiencing symptoms of a myelopathy. Men diagnosed at a later age generally have a more benign disease course with normal adrenal function [33]. This group is biochemically identifiable as shown by a recent study on lipidomic profiles [10]. Another difference between age groups is that, although SF‐36 subscores were lowest in the oldest patients, patients in the middle age group visited a psychologist or psychiatrist in the past year more frequently than patients in other age categories. These findings indicate that greater attention should be directed toward addressing psychological complaints, particularly in older patients. In contrast to urinary incontinence, fecal incontinence was present at about the same level in older patients as in younger patients. This suggests that reduced anal sphincter control can be an early sign of ALD and that vulnerability generally does not increase with age.

Strengths of this study are the large number of participants and the standardized, prospective and long‐term follow‐up. Limitations include that data was collected over time by five different physicians. Although instrument guidelines were used and quality checks were performed, some inter‐rater variability may still be present. Furthermore, The Dutch ALD cohort reflects the results of a single center. It is unclear how well the patients included in this study compare to other populations. The establishment of an international registry would allow for stronger conclusions on generalizability of these results. Last, 19% of participants dropped out of this study for various reasons (not specifically ALD or disease severity related). One individual experienced invalidating symptoms related to ALD, which prevented him from visiting the hospital. It is possible that the six patients that were lost to follow‐up may have had similar reasons for dropping out. This small number of individuals will likely have had little effect on the analyses and conclusions.

Our findings demonstrate that the EDSS, SSPROM and 6MWT can track disease progression, exhibiting near‐linear trends over time. However, observed changes are small and clinical trials using these outcome measures require long follow‐up and many patients [34]. If these outcome measures are used, it is necessary to include those with EDSS > 2.5 at baseline as only these patients will show changes over time. Future longitudinal studies should replicate this finding. It is not likely that the underlying disease progression (i.e., axonal loss in the long tracts of the spinal cords) is different between this groups, but that due to limitations of the used outcome measures (i.e., floor effect) change is not detectable in the EDSS < 2.5 group. New (surrogate) outcome measures that are more sensitive to small changes in disease progression are needed and are not (or less) susceptible to floor and ceiling effects are needed so that a wider range of patients can be included in future clinical trials. In recent years, there have been promising results in this field (for instance body sway) and it is imperative to further investigate their practical application [14, 15, 16, 17, 35].

## Author Contributions

**Hemmo A. F. Yska:** drafting of the manuscript; acquisition of data; analysis and interpretation of data. **Marije Voermans:** acquisition of data, revision of the manuscript. **Eda Kabak:** acquisition of data, revision of the manuscript. **Marc Engelen:** revision of the manuscript; study concept and design.

## Ethics Statement

All procedures followed were in accordance with the ethical standards of the responsible committee on human experimentation (institutional and national) and with the Helsinki Declaration of 1975, as revised in 2000. Informed consent was obtained from all patients for being included in the study. Ethical approval for this study was given by the local medical ethical review committee of the Amsterdam UMC.

## Conflicts of Interest

M. Engelen has received research support from Minoryx, Autobahn Therapeutics, BlueBirdBio and SwanBio; consultation fees from Minoryx, BlueBirdBio, Autobahn Therapeutics and Poxel.

## Supporting information


**Table S1.** Background at baseline.

## Data Availability

The data that support the findings of this study are available from the corresponding author upon reasonable request.
