# Supplementary material for: Progression of Spinal Cord Disease in Adult Men With Adrenoleukodystrophy
Source: J Inherit Metab Dis. 2025 Jan 7;48(1):e12845. doi: 10.1002/jimd.12845 (PMC11706703; doi:10.1002/jimd.12845)
Supplement: Supplementary file 1 — Table S1. Background at baseline. [file JIMD-48-0-s001.docx]

**Supplementary table 1: Background at baseline**

|  | EDSS ≤ 2,5 | | EDSS > 2,5 | |
| --- | --- | --- | --- | --- |
| General | **n** |  | **n** |  |
| Age (years) | 28 | 27.9 (11.7) | 51 | 48.8 (14.1) |
| Male balding | 28 | 14 (50%) | 50 | 44 (88%) |
| Glucocorticoid insufficiency | 28 | 19 (68%) | 51 | 21 (41%) |
| Mineralocorticoid insufficiency | 28 | 8 (29%) | 51 | 11 (22%) |
| Myelopathy | 28 | 7 (25%) | 51 | 50 (98%) |
| Visited psychologist in past year | 26 | 7 (27%) | 50 | 8 (16%) |
| Neurological examination |  |  |  |  |
| Pathological reflexes | 28 | 4 (14%) | 51 | 28 (55%) |
| Decreased strength (MRC<5) | 28 | 0 (0%) | 51 | 30 (59%) |
| Leg spasticity | 28 | 2 (7%) | 51 | 29 (57%) |
| Decreased propriocepsis | 25 | 1 (4%) | 47 | 31 (66%) |
| Decreased warm-cold discrimination sense | 25 | 2 (8%) | 38 | 19 (50%) |
| Postural instability | 28 | 1 (4%) | 49 | 45 (92%) |
| Degree of disability |  |  |  |  |
| Gait disorder | 28 | 3 (11%) | 51 | 44 (86%) |
| Falling/tripping | 28 | 2 (7%) | 51 | 42 (82%) |
| Walking with aid | Not applicable | | 51 | 3 (6%) |
| Use of wheelchair | Not applicable | | 51 | 1 (2%) |
| Independence in daily activities | 28 | 27 (96%) | 51 | 1 (2%) |
| Urogenital issues |  |  |  |  |
| Incontinence urine | 27 | 0 (0%) | 51 | 26 (51%) |
| Libido problems | 18 | 3 (17%) | 41 | 19 (46%) |
| Erectile dysfunction | 18 | 1 (6%) | 44 | 27 (61%) |
| Incontinence feces | 28 | 0 (0%) | 51 | 16 (31%) |
| Quantitative measures |  |  |  |  |
| EDSS | 28 | 0.8 (0.9) | 51 | 4.4 (1.3) |
| SSPROM | 27 | 98.2 (3.6) | 51 | 80.7 (8.4) |
| 6MWT (meters) | 24 | 638.8 (117.6) | 38 | 433.2 (123.1) |
| TUG (seconds) | 12 | 6.6 (2.3) | 17 | 10.9 (3.5) |
| SF36 physical functioning | 23 | 92.0 (13.7) | 44 | 52.7 (26.5) |
| SF36 physical health | 24 | 88.5 (27.6) | 48 | 62.0 (43.8) |
| SF36 emotional problems | 25 | 91.0 (26.4) | 48 | 77.8 (37.9) |
| SF36 energy/fatigue | 25 | 65.4 (18.8) | 48 | 52.4 (19.6) |
| SF36 well-being | 25 | 75.2 (19.5) | 48 | 63.1 (23.8) |
| SF36 social functioning | 25 | 83.6 (19.5) | 48 | 62.0 (22.5) |
| SF36 pain | 25 | 86.6 (17.9) | 48 | 73.6 (24.6) |
| SF36 average health | 25 | 66.6 (18.8) | 48 | 50.1 (19.1) |
| ICIQ-MLUTS- voiding | 21 | 3.0 (3.0) | 46 | 8.4 (5.2) |
| ICIQ-MLUTS-incontinence | 21 | 1.3 (1.5) | 46 | 5.4 (4.3) |
| Vibration sense hallux | 27 | 6.5 (2.0) | 43 | 1.1 (1.8) |

Data is presented in two EDSS categories as mean and (standard deviation) or as number of patients and (percentage). EDSS: Expanded Disability Scoring Scale; SSPROM: Severity Score System for Progressive Myelopathy; 6MWT: 6-minute walking test; TUG: timed-up-and-go; SF-36: Short-Form health survey; ICIQ-MLUTS: International Consultation on Incontinence Questionnaire Male Lower Urinary Tract Symptoms Module.
